# Human Platelet Antigen Genotyping and Expression of CD109 (Human Platelet Antigen 15) mRNA in Various Human Cell Types

**DOI:** 10.1155/2013/946403

**Published:** 2013-02-12

**Authors:** Sang Mee Hwang, Mi Jung Kim, Ho Eun Chang, Yun Ji Hong, Taek Soo Kim, Eun Young Song, Kyoung Un Park, Junghan Song, Kyou-Sup Han

**Affiliations:** ^1^Department of Laboratory Medicine, Seoul National University College of Medicine, Seoul 110-744, Republic of Korea; ^2^Department of Laboratory Medicine, Seoul National University Bundang Hospital, 173-82 Gumiro, Bundanggu, Gyeonggido, Seongnam 463-707, Republic of Korea

## Abstract

CD109 gene encodes a glycosylphosphatidylinositol-linked glycoprotein found in a subset of platelets and endothelial cell, and human platelet antigen (HPA) 15 is found on CD109. We evaluated the HPA genotype and/or the CD109 mRNA expression on two peripheral blood stem cells (PBSC), two peripheral bloods (PB), 12 granulocyte products, natural killer (NK)-92, B-lymphocyte (CO88BV59-1), K-562 leukemia cell line, human embryonic stem cell (hESC), and human fibroblasts (HF). HPA genotyping was performed by SNaPshot assay and CD109 mRNA expression was evaluated by real-time PCR with SYBR green and melting curve analysis. Genotype HPA-15a/-15a was found in PBSC#1 and two granulocyte products, and HPA-15a/-15b was found in PBSC#2, eight granulocyte products, NK-92, K-562, hESC, and HF, and HPA-15b/-15b was found in two granulocyte products. CD109 mRNA expression was highly increased in HF and increased in CD34+ and CD34− PBSCs and some granulocyte products, compared to the PB. However, the increase of expression level varied among the PBSC and granulocyte products. The CD109 mRNA expression of NK-92, K-562, hESC, and CO 88BV59-1 was not detected. HPA genotype was evaluated in various cells and the expression of CD109, which contains HPA 15, was different among cell lines and high in HF and PBSCs.

## 1. Introduction

The CD109 gene encodes a glycosylphosphatidylinositol linked glycoprotein, that is, a member of the alpha2-macroglobulin/complement family of thioester-containing proteins [1]. CD109 is expressed on platelets, activated T-cells, and endothelial cells [2] and has gained clinical attention due to the association of antibodies to CD109 with alloimmune thrombocytopenia and posttransfusion purpura [3]. Depending on a single nucleotide polymorphism at nucleotide of coding CD109 gene, human platelet antigen (HPA)-15a, and HPA-15b alleles are defined [4], and anti-HPA-15 antibodies may develop and cause clinical consequences. Moreover, CD109 mRNA transcript has been studied in various cancers and is found to be highly expressed in squamous cell carcinomas and melanomas [5–7]. CD109 has been reported as a TGF-*β* coreceptor with high affinity for the TGF-*β* subtype and inhibiting TGF-*β* signaling in vitro, thus has been studied as a therapeutic target for diseases in which TGF-*β* may play a pathophysiological role and a key to elucidate pathogenesis of certain cancers [6, 8]. Thus, CD109 mRNA expression has been studied in some cells and tissues [9–11]. However, the studies of the CD109 mRNA expression have been limited to few cell types thus we have evaluated mRNA expression in a wider variety of cell types and cell lineages including common cell lines. The cell types or cell lineages included in this study are peripheral blood stem cells (PBSC), granulocyte products, natural killer (NK)-92 cell line, B-lymphocyte cell line (CO 88BV-59-1), K-562 leukemia cell line, human embryonic stem cell (hESC), and human fibroblasts (HF). In addition, the genotype of different HPAs including the HPA 15, which is included in the CD109, was also characterized.

## 2. Materials and Methods

### 2.1. Materials

PBSC or granulocytes were collected by Cobe Spectra (Terumo BCT, Lakewood, USA) in two patients for PBSC and 12 patients for granulocyte products with informed consent. CliniMACS Cell separation system (Miltenyi Biotec, Bergisch Gladbach, Germany) was used to obtain CD34 enriched and depleted population of PBSC. PB prior to PBSC or granulocyte collection was also collected from donors.

Three cell lines including NK-92, B-lymphocyte CO88BV59-1, and K-562 leukemia cell lines were used in this study. Human NK-92 cell line was purchased from the American Type Culture Collection (ATCC CRL-2407, Rockville, MD) and maintained in alpha MEM medium supplemented with 12.5% horse serum, 12.5% fetal bovine serum (FBS), 0.2 mM myoinositol, 0.1 mM 2-mercaptoethanol, 0.02 mM folic acid, and recombinant human interleukin-2 (Proleukin, Prometheus, San Diego, CA). CO 88BV59-1 (CRL-10624, ATCC) and K-562 (ATCC) were maintained in RPMI-1640 (Invitrogen, Carlsbad, CA, USA), 10% FBS (Invitrogen).

Other cell types including hESC (SNUhES12 from Institute of Reproductive Medicine and Population in Seoul National University Medical Research Center) and HF (Modern Cell & Tissue Technology, Seoul, Korea) were maintained in RPMI-1640 with 10% FBS. Mouse embryonic fibroblast (MEF) (CF-1 MEF, Modern Cell & Tissue Technology) was also included as a negative control for PCR amplification. Different cells or cell lines were kept at 37°C, 5% CO_2_.

### 2.2. Human Platelet Antigen Genotyping

HPA genotyping was performed for HPA-1, -2, -3, -4, -5, -6, -7, -8, -9, -13, and -15 using SNaPshot assay. Excluding the B-lymphocyte CO 88BV59-1 cell line, all of the cells or cell lines were included in the HPA genotyping. DNA was extracted using QIAamp DNA Blood mini kit (Qiagen). The primers for amplification were used as previously described [12]. PCR was performed with 100 ng of DNA, 0.4 *μ*M for each primer, 0.625 U of *Taq* polymerase (Takara Bio Inc., Otsu, Japan), 10X buffer of 2.5 *μ*L, 2.0 *μ*L of 2.5 mM dNTPs, and 16.0 *μ*L of distilled water. Thermocycling was performed as follows. Initial denaturation was carried out at 95°C for 10 minutes, amplification for 35 cycles at 94°C for 30 seconds, 55°C for 30 seconds, and 72°C for 1 minute and extension at 72°C for 7 minutes. Amplified PCR products were purified using the ExoSAP purification kit (ExoSap-it, Affymetrix, Cleveland, OH, USA). SNaPshot analysis was performed using an ABI PRISM SNaPshot Multiplex kit (Applied Biosystems, Foster City, CA, USA). Two sets of SNaPshot reaction were performed. The first set included the reaction for HPA-1, -2, -3, -4, and -9, and the second reaction was for HPA-5, -6, -7, -8, -13, -15. The sequences of the SNaPshot primers are shown in Table 1. The multiplex SNaPshot reaction was performed in a final volume of 10 *μ*L, containing 50 ng of each template, 5.0 *μ*L of the SNaPshot multiplex ready reaction mix, and 4 *μ*M of each SNaPshot primers for HPA-1, -2, -3, -4, -5, -6, -7, -9, and 10 *μ*M of primers for HPA-8, -13, and -15. Cycling conditions were 25 cycles of 96°C for 10 seconds, 50°C for 5 seconds, and 60°C for 30 seconds. SNaPshot products were treated with shrimp alkaline phosphatase, separated using 0.15 *μ*L of GeneScan-120 LIZ size standard with a ABI PRISM 3100 Genetic Analyzer, and data were analyzed using the GeneMapper Analysis Software version 2.0 (Applied Biosystems).

### 2.3. CD109 mRNA Expression

RNA was isolated from all of the cells included in the study with High Pure RNA Isolation kit (Roche, Mannheim, Germany) and reverse transcribed (SuperScript III Reverse Transciptase, Invitrogen). cDNA was amplified using a real-time PCR with the Light Cycler FastStart DNA Master SYBR Green I kit (Roche) in a LightCycler 2.0 (Roche). The following primer sequences were used for detecting expression of CD109 mRNA relative to *β*_2_-microglobulin: the forward primer for CD109 was 5′-TAGCAGTCCACATGTCCGAAAGCA-3′ and the reverse primer was 5′-AACCAGTAGCCACCCAAGAAGTGA-3′ and the forward primer for *β*_2_-microglobulin was 5′-AGATGAGTATGCCTGCCGTGTGAA-3′ and the reverse primer was 5′-TGCGGCATCTTCAAACCTCCATGA-3′. PCR was performed with 2 *μ*L of cDNA, 0.2 *μ*M of each primer, 2 *μ*L of 10X mixture, and 12.8 *μ*L of distilled water. Thermocycling was performed as follows: initial denaturation was carried out at 94°C for 10 minutes, amplification for 40 cycles at 94°C for 10 seconds, 58°C for 10 seconds, and 72°C for 15 seconds. Melting curve analysis was performed for 1 cycle at 95°C for 5 seconds, 65°C for 30 seconds, and 99°C for 0 seconds with a ramp rate of 0.2°C per seconds and 40°C for 30 seconds. Expression of CD109 was compared using *β*_2_-microglobulin as the reference gene and the PB from donors of PBSC included in the study as the control. In case of cell lines, PB of PBSC#2 was used as a reference. The relative gene expression was calculated by the modified 2^−ΔΔCt^ method [13].

## 3. Result

### 3.1. Human Platelet Antigen Genotyping

The results of HPA-1, -2, -3, -4, -5, -6, -7, -8, -9, -13, and -15 genotyping of different cell lines and cells are shown in Table 2. For HPA-15, genotype HPA-15a/-15a was found in PBSC#1 and two granulocyte products, genotype HPA-15a/-15b was found in PBSC#2, eight granulocyte products, NK-92, K-562, hESC, and HF, and genotype HPA-15b/-15b was found in two granulocyte products.

### 3.2. CD109 mRNA Expression

**Figure 1:**
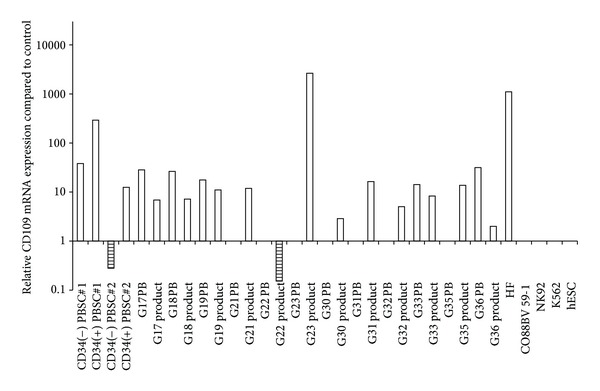
Comparison of CD109 mRNA expression results relative to the expression of those from peripheral blood (PB) of a PBSC donor. The expression of control PB is set as 1.0. The samples with no bar depict specimens with no detectable CD109 mRNA expression.

The expression of CD109 mRNA was detected in PB of PBSC donors, PBSC products including CD34 enriched and depleted populations, HF, PB of five granulocyte donors, and all of the granulocyte products. However, CD109 mRNA expression was not detected in the PB of seven granulocyte donors, NK-92, K-562, and CO 88BV59-1 cell lines (Figure 1).

The expressions of PBSCs were compared to the PB of donors prior to PBSC collection. CD34+ enriched population showed higher expression of CD109 mRNA compared to the product prior to enrichment. PBSC#1 products showed higher CD109 mRNA expression than PBSC#2 products. The CD34+ percentage and count of CD34+ enriched population was higher in PBSC#1 (86.7%, 741.1/*μ*L) than for PBSC#2 (74.4%, 355.5/*μ*L). The CD109 mRNA expression of other cell lines and cells were compared to the expression of the PB of PBSC donors. HF showed markedly increased CD109 mRNA expression. For granulocyte products, only PB of five donors showed detectable level of mRNA expression. However, all of the granulocyte products were detected with CD109 mRNA expression. Depending on the donors, the CD109 mRNA expression of the product was higher in PB prior to granulocyte collection in five donors but the expression was higher in the granulocyte product in the remaining seven donors.

## 4. Conclusion

Genotyping of human platelet antigen including HPA 15, which is located in CD109, was performed on different cells and cell lines. There have been no previous reports on the HPA genotype of cell lines, NK-92, CO 88BV59-1 B-lymphocyte, K-562 leukemia, cell lines, and cells including HF and hESC. K-562 is a cell line made from a chronic myeloid leukemia patient [14] and showed HPA-1a/HPA-1b genotype, which is found rarely in Koreans [15]. All the other cells showed genotypes that were frequently found in Koreans [15, 16].

The expression of CD109 mRNA expression was also determined in different cell types and cell lines. Consistent to the previous reports on expression of CD109 on CD34+ hematopoietic stem cells [17], we were able to find high expression of CD109 mRNA on CD34+ enriched population compared to the PB prior to PBSC collection. However, the CD34 depleted population also showed detectable CD109 mRNA expression but lower than PB of PBSC donors. This may be because PB of PBSC donors have significantly high level of hematopoietic stem cells recruited to the PB with prior treatment of granulocyte-colony stimulating factors. Thus, the relative mRNA expression may be different when compared to the PB from normal donors without prior G-CSF treatment. Interestingly, CD34+ products of a donor with higher percentage and count of CD34 enriched population showed higher expression of CD109 mRNA. Further analysis of CD34+ cells in different products may explain the CD109 mRNA expression level as well. HF, which is also known to have CD109 protein expression [18, 19] showed very high expression of CD109 mRNA. In a previous study, the level of CD109 mRNA expression was compared with normal fibroblasts and scleroderma skin fibroblasts and showed no significant difference [19]. In our study, the mRNA expression of HF was compared to the PB of a PBSC donor and was very high. The CD109 mRNA expression of NK-92, CO 88BV 59-1, and K-562 cell lines were not detected. There have been reports on the expression of CD109 on activated T-lymphocytes [20, 21], but no reports have been made on B-lymphocytes and NK cells.

This was the first study to genotype HPA in various known cell lines and different cells. Moreover, the mRNA expression of CD109 studied in each cell types and was found to be high in PBSCs, some granulocyte products and HF compared to the PB but was not detected in NK cell line, B-lymphocyte cell line, and hESC.

## Figures and Tables

**Table 1:** SNaPshot primer sequences.

HPA type	Sense primer	Fragment size (bp)
Set 1		
HPA-1	5′-ggtcacagcgaggtgagccc-3′	20
HPA-2	5′-N_2_gatgcccccagggctcctga-3′	30
HPA-3	5′-N_5_aatgggggaggggctgggg-3′	39
HPA-9	5′-N_7_cactcctttgcccccccag-3′	47
HPA-4	5′-N_9_caagctggccacccagatgc-3′	56
Set 2		
HPA-5	5′-gagtctacctgtttactatcaaa-3′	23
HPA-6	5′-N_3_caggacgaatgcagccccc-3′	31
HPA-7	5′-N_5_aggccaaggtgcgaggctgt-3′	40
HPA-8	5′-N_7_atacctgcaaccgttactgc-3′	48
HPA-13	5′-N_9_aaggttaacattttcagtaa-3′	56
HPA-15	5′-N_11_caaattcttggtaaatcctg-3′	64

N: Poly(dGACT) tail.

**Table 2:** Human platelet antigen genotype results by SNaPshot assay.

Cell types	HPA-1	HPA-2	HPA-3	HPA-4	HPA-5	HPA-6	HPA-7	HPA-8	HPA-9	HPA-13	HPA-15
	(176T > C)	(482C > T)	(2621T > G)	(506G > A)	(1600G > A)	(1544G > A)	(1297C > G)	(1984C > T)	(2602G > A)	(2483C > T)	(2108C > A)
NK-92	T/T	C/C	T/G	G/G	G/A	G/G	C/C	C/C	G/G	C/C	C/A
K-562	C/T	C/C	T/G	G/G	G/G	G/G	C/C	C/C	G/G	C/C	C/A
hESC	T/T	C/C	T/G	G/G	G/G	G/G	C/C	C/C	G/G	C/C	C/A
HF	T/T	C/C	T/G	G/G	G/G	G/A	C/C	C/C	G/G	C/C	C/A
PBSC product #1	T/T	C/C	T/G	G/G	G/G	G/G	C/C	C/C	G/G	C/C	C/A
PBSC product #2	T/T	C/C	T/G	G/G	G/G	G/G	C/C	C/C	G/G	C/C	C/C
Granulocyte G17	T/T	C/C	T/G	G/G	G/A	G/G	C/C	C/C	G/G	C/C	C/A
Granulocyte G18	T/T	C/C	T/G	G/G	G/G	G/G	C/C	C/C	G/G	C/C	C/A
Granulocyte G19	T/T	C/C	T/T	G/G	G/G	G/G	C/C	C/C	G/G	C/C	C/A
Granulocyte G21	T/T	C/C	T/T	G/G	G/G	G/G	C/C	C/C	G/G	C/C	C/A
Granulocyte G22	T/T	C/T	T/T	G/G	G/G	G/G	C/C	C/C	G/G	C/C	C/C
Granulocyte G23	T/T	C/T	T/T	G/G	G/G	G/G	C/C	C/C	G/G	C/C	A/A
Granulocyte G30	T/T	C/C	T/T	G/G	G/A	G/G	C/C	C/C	G/G	C/C	C/C
Granulocyte G31	T/T	C/C	T/G	G/G	G/G	G/G	C/C	C/C	G/G	C/C	C/A
Granulocyte G32	T/T	C/T	T/G	G/G	G/A	G/G	C/C	C/C	G/G	C/C	C/A
Granulocyte G33	T/T	C/T	T/G	G/G	G/G	G/G	C/C	C/C	G/G	C/C	A/A
Granulocyte G35	T/T	C/C	T/G	G/G	G/G	G/G	C/C	C/C	G/G	C/C	C/A
Granulocyte G36	T/T	C/T	T/T	G/G	G/G	G/G	C/C	C/C	G/G	C/C	C/A
